# Supplementary material for: Transcriptomic Insights into Mechanisms of Early Seed Maturation in the Garden Pea (Pisum sativum L.)
Source: Cells. 2020 Mar 23;9(3):779. doi: 10.3390/cells9030779 (PMC7140803; doi:10.3390/cells9030779)

Zhewan−1(25) vs Zhon

Global Summary

%DE = 0.85

genes with fdr < 0.2 = 1798 (723 + / 1075 −)

genes with fdr < 0.1 = 1598 (656 + / 942 −)

genes with fdr < 0.05 = 1453 (616 + / 837 −)

genes with fdr < 0.01 = 955 (423 + / 532 −)

genes in genesets = 0

<FC> = 0

<t−score> = −0.5

<p−value> = 0.01

<fdr> = 0.15

Global Genelist

Rank	ID	log(FC)	p−value	fdr / Metagene	Description
1	TRINITY_DN4	−3.18	7e−10	0.0000008 x 1	
2	TRINITY_DN1	0.02	8e−09	0.00000088 x 17	
3	TRINITY_DN1	0.02	8e−09	0.00000087 x 1	
4	TRINITY_DN2	0.05	1e−08	0.00000081 x 9	
5	TRINITY_DN7	0.66	1e−08	0.00000080 x 25	
6	TRINITY_DN1	1.43	1e−08	0.00000080 x 30	
7	TRINITY_DN2	0.03	2e−08	0.00000199 x 17	
8	TRINITY_DN2	0.02	2e−08	0.00000318 x 18	
9	TRINITY_DN1	0.71	4e−08	0.0000035 x 6	
10	TRINITY_DN6	2.22	5e−08	0.00000330 x 30	
11	TRINITY_DN2	1.84	5e−08	0.00000330 x 29	
12	TRINITY_DN1	−0.74	7e−08	0.00000325 x 30	
13	TRINITY_DN1	−0.1	8e−08	0.00000313 x 25	
14	TRINITY_DN3	−0.23	9e−08	0.0000031 x 27	
15	TRINITY_DN4	0.02	9e−08	0.00001145 x 9	
16	TRINITY_DN5	−3.01	1e−07	0.0000114 x 30	
17	TRINITY_DN2	0.13	2e−07	0.00001149 x 17	
18	TRINITY_DN4	−0.1	2e−07	0.0000118 x 5	
19	TRINITY_DN1	0.03	3e−07	0.00001148 x 16	
20	TRINITY_DN1	−1.76	3e−07	0.0000114 x 30	

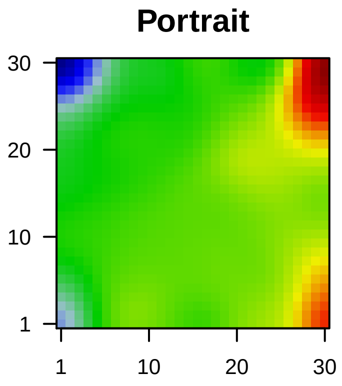

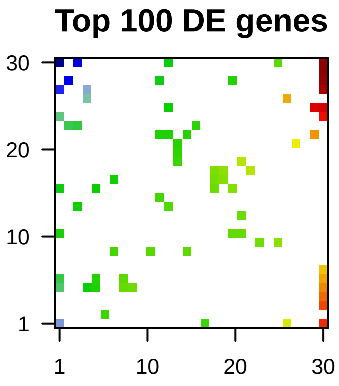

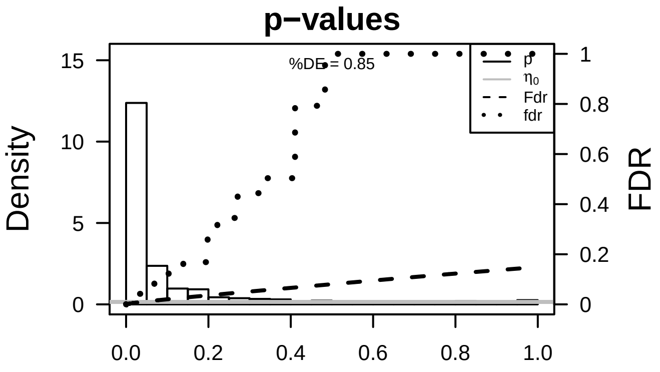

Supplement: Supplementary file 1 [file cells-09-00779-s001.zip › Supplementary materials/FileS4.zip!/FileS3/TFs - Results/Summary Sheets - Differences/Reports/Zhewan.1.25..vs.Zhongwan.6.25..pdf]
